# Skeletal Muscle Radio-Density Is an Independent Predictor of Response and Outcomes in Follicular Lymphoma Treated with Chemoimmunotherapy

**DOI:** 10.1371/journal.pone.0127589

**Published:** 2015-06-01

**Authors:** Michael P. Chu, Jessica Lieffers, Sunita Ghosh, Andrew R. Belch, Neil S. Chua, Amelie Fontaine, Randeep Sangha, A. Robert Turner, Vickie E. Baracos, Michael B. Sawyer

**Affiliations:** Department of Medical Oncology, Cross Cancer Institute, University of Alberta, Edmonton, Alberta, Canada; Northwestern University Feinberg School of Medicine, UNITED STATES

## Abstract

Skeletal muscle radio-density (SMD) measures muscle radiation attenuation (in Hounsfield Units, HU) on computed tomography (CT) scans. Low SMD is prognostic of poor survival in melanoma, however its significance is unknown for hematologic malignancies. We performed a single institution, retrospective review of all follicular lymphoma (FL) patients who received chemoimmunotherapy from 2004–2009. Patient demographics, FL International Prognostic Index 1 (FLIPI-1), progression free (PFS) and overall survival (OS) were collected as primary endpoints. Objective response rates (ORR) were secondary. SMD was calculated using pre-treatment CT scans. In 145 patients reviewed, median values were age 59, FLIPI-1 of 2, stage III, and 8 chemoimmunotherapy cycles received. Median PFS for those with low SMD (<36.6 and <33.1 HU for patients with BMI ≤ 25 and > 25 kg/m^2^, respectively) compared to those with high SMD was profoundly worse, 69.6 vs. 106.7 months (hazard ratio [HR] 1.85; *p* = 0.01), respectively. Median OS was not reached in patients with high SMD vs. 92.7 months in low SMD patients (HR 4.02; *p* = 0.0002). Multivariate analysis supported lower SMD’s OS detriment (HR = 3.40; *p* = 0.002) independent of FLIPI-1 (HR 1.46–2.76, *p* = 0.05) or gender. Low SMD predicted lower ORR, 83 vs. 96% (*p* = 0.01). SMD predicts survival independent of FLIPI-1 and potentially chemoimmunotherapy response. SMD is an inexpensive and powerful tool that can complement FLIPI-1.

## Introduction

Weight loss is considered a poor prognostic indicator across many diseases. More recently, work into what contributes to this poor prognosis has led to investigation of body composition, a study of the proportion of lean and fat tissues in the human body. Though severe loss of skeletal muscle mass (sarcopenia) is prognostic in oncology [1–4], skeletal muscle (radio)-density (SMD) has also been found to be prognostic across multiple tumor subtypes [4–6]. SMD is average radiation attenuation of tissue in Hounsfield Units (HU) measured in computed tomography (CT) imaging. Muscle is usually quantified within a radiation attenuation range of -30 to 150 HU, but normal muscle in healthy young individuals typically has a peak radiation attenuation of 50 ± 10 HU. Muscle with attenuation < 30 HU is considered less functional and found in disease states such as myopathies and type II diabetes [7, 8]. Low muscle attenuation, or low SMD, has an appearance of ectopic fat infiltration. This phenomenon has been assessed on pre-treatment CT images and was recently found to be predictive of outcomes in metastatic renal cell cancer and melanoma independent of targeted therapy use [5, 6]. Inflammation is a proposed link between development of intramuscular adipose tissue and diabetes [7], but its underlying pathology in oncology has not been established. However, the value of what constitutes low SMD is dependent on body mass index (BMI) given that fatty infiltration of muscle is more prevalent in overweight and obese individuals [4].

While body composition analysis is a relatively new approach, the original Hodgkin Lymphoma Ann Arbor staging system recognized weight loss as prognostic and included it as a B symptom. It was subsequently extrapolated to Non-Hodgkin Lymphomas such as follicular lymphoma (FL) [9]. Both aggressive and indolent lymphoma treatment has changed dramatically with rituximab, a monoclonal antibody targeting CD20 [10–12]. While the most effective chemotherapy with rituximab was a point of contention prior to the STiL trial [13], there is a significant improvement in both progression free survival (PFS) and overall survival (OS) in FL patients treated with rituximab-chemotherapy regimens. The RICOVER60 trial studied rituximab’s use with cytotoxic chemotherapy in diffuse large B-cell lymphoma. In subgroup analysis though, males did not have as large of an improvement in both PFS and OS compared to females [14]. Based on pharmacokinetic studies, it was thought that males’ higher weight increased rituximab’s clearance and volume of distribution, decreasing its efficacy in males. After its incorporation as a standard therapy, retrospective data still confirms gender disparity [15]. Gender effects in FL were not thought to be as significant based on retrospective data. However, a recent prospective study by Jager *et al*. [16] showed females have significantly higher rituximab serum levels compared to males. Females’ higher levels in turn resulted in improved response and PFS to chemoimmunotherapy.

We speculate that gender-based response to chemoimmunotherapy may actually reflect a variation in muscle mass. Females are inherently less muscular than males, and therefore have less skeletal muscle mass than males for any given height and weight. Elderly lymphoma patients also have a poorer prognosis based on the revised International Prognostic Index score (R-IPI) [17]; we theorized that this is related to the generally lower skeletal muscle mass in this population. Lanic *et al*. [18] have examined this retrospectively and demonstrated that sarcopenia is prognostic in elderly patients treated for aggressive lymphoma independent of R-IPI. However, a discrepancy is inherent with using age as a cut-off given that the Lanic group used an age of 70 years or older to define their elderly cohort while the R-IPI score uses 60 years of age. Perhaps a more unifying assessment that is less dependent on age and gender may in fact be SMD. By virtue of measuring radiation attenuation, SMD does not differ between genders as compared to muscle mass [4]. Whether these hypotheses can be extrapolated to FL is unclear.

The goal of this study was to explore whether underlying gender- and age-based outcomes in FL were related to SMD and sarcopenia.

## Methods

### Patients

FL patients seen at a regional cancer centre treating all patients from Northern Alberta, Canada (catchment population of > 1.8 million) treated with rituximab-based chemotherapy from 2004 to 2009 were reviewed. Collected patient demographics included age, stage, FL International Prognostic Index-1 score (FLIPI-1) at diagnosis, gender, height, weight, and performance status. The chemotherapy regimen they received, number of cycles received, and best response to first-line therapy was then documented. PFS and OS were collected as primary endpoints and calculated from time of first treatment. Best radiographic response to first-line chemoimmunotherapy was collected as a secondary endpoint.

### Body Composition Analysis

Height and weight at first cycle of treatment was documented. Body composition was assessed using patients’ pre-treatment, staging CT scans. To be included in review, CT scans must have been within 30 days prior to beginning therapy. CT scan reviewers were trained how to perform CT scan analysis using the Slice-O-Matic (version 4.3, TomoVision, Magog, Quebec, Canada) software by an expert trainer and then tested with standard exam so that the trainees’ results were within 1.5% of established parameters from test CT scans. To ensure image quality, CT scanner calibration was performed first daily at start up with using air in the CT scanner gantry, then dynamically during scanning for individual patients using air as a negative control. Therefore, though CT tube current may fluctuate between patients, the variability is minimal yielding the following CT parameters for each patient: contrast enhanced or unenhanced, 5-mm slice thickness, 120 kVp, and nearly constant 290 mA. Two adjacent images at the L3 vertebral body level were used to measure total muscle surface area (cm^2^) and averaged. This vertebral landmark is chosen based on its linear correlation to total body LBM [19, 20]. Muscles were quantified within a HU range of -29 to 150 HU using Slice-O-Matic software. Measurements were normalized to patient’s height in meters squared and expressed as skeletal muscle index (cm^2^/m^2^).

SMD was expressed as mean muscle radiation attenuation (HU) using Slice-O-Matic where the tissue cross-sectional area was assessed between -29 to +150 HU [21].

### Statistical Analysis

As an exploratory retrospective cohort, a specific low level of skeletal muscle index and SMD was hypothesized to be associated with reduced PFS and OS, however the specific cut-off value is unknown. Therefore, pre-planned analyses sought to determine this level of skeletal muscle index and SMD. Though less data is available for SMD assessment, Martin *et al*. have established a cut off of 41 HU and 33 HU in non-overweight (BMI ≤ 25 kg/m^2^) and overweight (BMI > 25 kg/m^2^) patients, respectively [4]. Our cohort was analyzed for SMD significance dividing into the following groups: 1) above and below an SMD of 41 HU and 33 HU in non-overweight and overweight patients; and 2) cut-point analysis. Skeletal muscle index was compared by dividing into the following groups: 1) above and below 55.8 cm^2^/m^2^ in males and 38.9 cm^2^/m^2^ for females as outlined by Lanic *et al*. [18]; 2) above and below levels established in obese patients (52.4 cm^2^/m^2^ in males and 38.5 cm^2^/m^2^ in females) as outlined by Prado *et al*. [2]; 3) around levels established in non-overweight patients with solid organ malignancies (43 cm^2^/m^2^ in males and 41 cm^2^/m^2^ in females) [4]; and 4) cut-point analysis.

In both SMD and skeletal muscle index cut-point analyses, the continuous variable was dichotomized using minimum *p*-value method [22]. The continuous variable is divided into different cutpoints and the cutpoint that provides the maximum chi-square or minimum *p*-value is chosen to be the point for dichotomizing the continuous variable.

Median PFS and OS were estimated using Kaplan-Meier methods for each of these groups. Survival and objective response rates (ORR) by Cheson criteria [23] were then compared by log-rank test and multivariate Cox proportional hazards ratios using the same covariates. Statistical analysis was done through Statistical Analysis System (SAS, version 9.3 from SAS Institute Incorporated, Cary, North Carolina).

### Ethics Statement

As a retrospective study, patient consent was not obtained because this study presented minimal risk to patients or patient identifiers and data was analyzed anonymously; institutional approval was granted by the Alberta Cancer Research Ethics Committee.

## Results

### Patients

Between January 2004 and December 2009, 155 FL patients received therapy at our institution and 145 were deemed eligible for review. Ten excluded patients did not have a CT within 30 days of starting therapy. Median age at diagnosis was 57 years (range 29–83) while median age at time of treatment was 59 years (range 29–84). There were 80 (55%) male patients and 65 (45%) female. The majority had either stage III (34%) or stage IV (45%) disease. FLIPI-1 scores were predominantly 1 (23%), 2 (32%) and 3 (23%). The majority had grade 1–2 disease (87%) with only one patient with grade 3B disease not treated as a more aggressive NHL variant. All patients received rituximab-based systemic therapy with all patients receiving R-CVP (rituximab, cyclophosphamide, vincristine, and prednisone). The median number of treatments was 8 cycles (range 1–8). A majority (n = 87, 60%) would go onto receive maintenance rituximab following completion of chemoimmunotherapy. A summary of patient demographics is found in Table 1. Together, median PFS and OS were 94.4 months and not reached, respectively.

**Table 1:** Patient demographics and response to therapy separated by Skeletal Muscle Density (SMD) and Body Mass Index (BMI) as defined by low (≤25 kg/m^2^) or high (>25 kg/m^2^).

Demographic	All (145)	Low SMD. Low BMI (13)	Low SMD, High BMI (46)	High SMD, Low BMI (27)	High SMD, High BMI (59)	*p*-value (low vs. high SMD)
Age at diagnosis, median (years)	57 (29–83)	67	61	57	52	<0.0001
Age at time of treatment, median (years)	59 (30–84)	69	62	59	53	<0.0001
Gender, #						0.21
Male	80 (55%)	5 (39%)	24 (52%)	12 (44%)	38 (64%)	
Female	65 (45%)	8 (62%)	22 (48%)	15 (56%)	21 (36%)	
Body weight, median (kg)	80.0	64.6	89.4	64.3	83.0	<0.0001
Body mass index, median (kg/m^2^)	27.7	24.1	30.9	22.8	28.3	<0.0001
Histologic Grade, #						0.72
1–2	126 (87%)	9 (69%)	41 (89%)	23 (85%)	53 (90%)	
3A	18 (12%)	4 (31%)	5 (11%)	3 (11%)	6 (10%)	
3B	1 (1%)	0	0	1 (4%)	0	
Stage, #						0.68
I	10 (7%)	1 (8%)	1 (2%)	2 (7%)	6 (10%)	
II	20 (14%)	2 (15%)	8 (17%)	3 (11%)	7 (12%)	
III	50 (34%)	4 (31%)	15 (33%)	9 (33%)	22 (37%)	
IV	65 (45%)	6 (46%)	22 (48%)	13 (48%)	25 (42%)	
FLIPI-1, #						0.01
0	11 (8%)	1 (8%)	3 (7%)	1 (4%)	7 (12%)	
1	34 (23%)	3 (23%)	11 (24%)	6 (22%)	14 (24%)	
2	46 (32%)	1 (8%)	10 (22%)	11 (41%)	23 (40%)	
3	33(23%)	2 (15%)	13 (28%)	8 (30%)	10 (17%)	
4	18 (12%)	4 (31%)	9 (20%)	1 (4%)	4 (7%)	
5	3 (2%)	2 (15%)	0	0	1 (2%)	
Chemoimmunotherapy cycles received, median	8	8	8	8	8	0.29
Best response to chemoimmunotherapy, #						0.01
Complete response	73 (50%)	7 (54%)	18 (39%)	15 (56%)	33 (56%)	
Partial response	58 (40%)	6 (46%)	18 (39%)	12 (44%)	22 (37%)	
Stable disease	4 (3%)	0	3 (7%)	0	1 (2%)	
Progressive disease	10 (7%)	0	7 (15%)	0	3 (5%)	
Received maintenance, #	87 (60%)	8 (62%)	32 (70%)	11 (41%)	36 (61%)	0.11
Required at least 1 cycle delayed, #	49 (34%)	6 (46%)	12 (26%)	11 (41%)	20 (34%)	0.49
CT Measures						
Mean skeletal muscle area ± standard error (cm^2^)	147.5 ± 3.0	125.4 ± 6.7	150.1 ± 5.3	134.7 ± 6.7	156.0 ± 4.7	0.008
Mean skeletal muscle index ± standard error (cm^2^/m^2^)	50.7 ± 0.8	45.9 ± 1.6	51.8 ± 1.4	45.0 ± 1.5	53.4 ± 1.2	0.0004
Mean SMD ± standard error (HU)	35.3 ± 0.8	29.9 ± 2.0	26.6 ± 0.8	44.6 ± 1.4	38.9 ± 0.6	<0.0001

### Skeletal Muscle Density

Median SMD for the entire group was 35.6 HU. SMD was strongly associated with outcomes. Using Martin *et al*. SMD cut-offs as established in solid organ malignancies, PFS in patients with low SMD was 71.7 months compared to those with high SMD at 91.2 months (HR 1.78, 95% CI 0.86–2.31, *p* = 0.18). Similarly, OS was 118.3 months *vs*. not reached for patients below and above Martin *et al*. parameters, respectively (HR 3.33, 95% CI 1.56–7.13, *p* = 0.002).

However, cut-point analysis revealed a more substantial difference in survival outcomes with SMD at 33.1 and 36.6 HU for non-overweight and overweight patients, respectively. Of those identified as having low SMD (n = 59, 40.7%), a statistically significant higher proportion had advanced age (≥ 60 years, n = 39, 66.1% vs. n = 31, 36.1%) and advanced FLIPI-1 scores (Table 1). PFS in patients with higher SMD was 106.7 months compared to the lower SMD group at 69.6 months (HR 1.85, 95% CI 1.13–3.03, *p* = 0.01). Similarly, OS in the higher SMD group was not reached *vs*. 92.7 months in the lower group (HR 4.02, 1.93–8.37, *p* = 0.0002).

**Fig 1:**
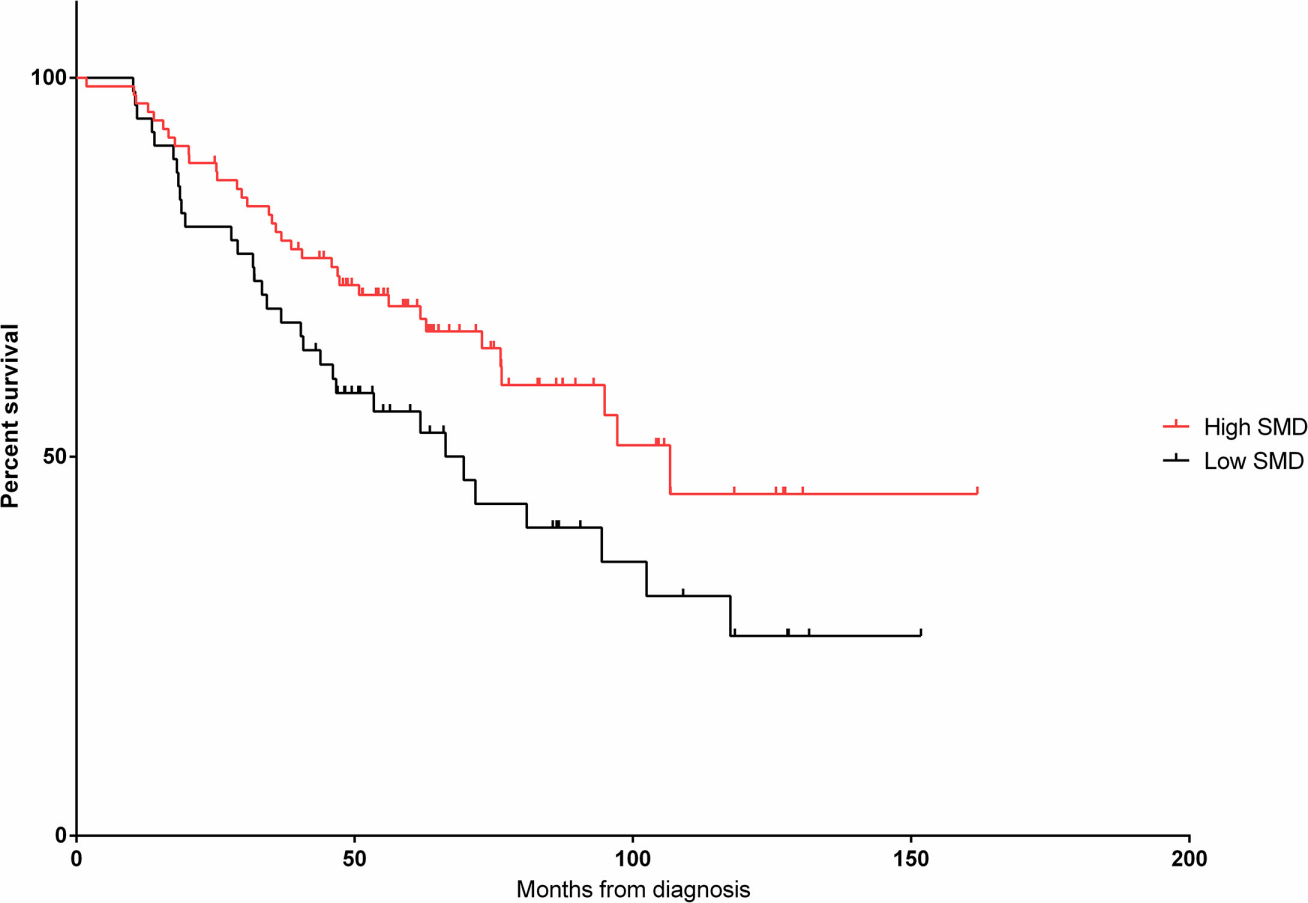
Progression Free Survival (PFS) along Skeletal Muscle Density (SMD) cut-point.

**Fig 2:**
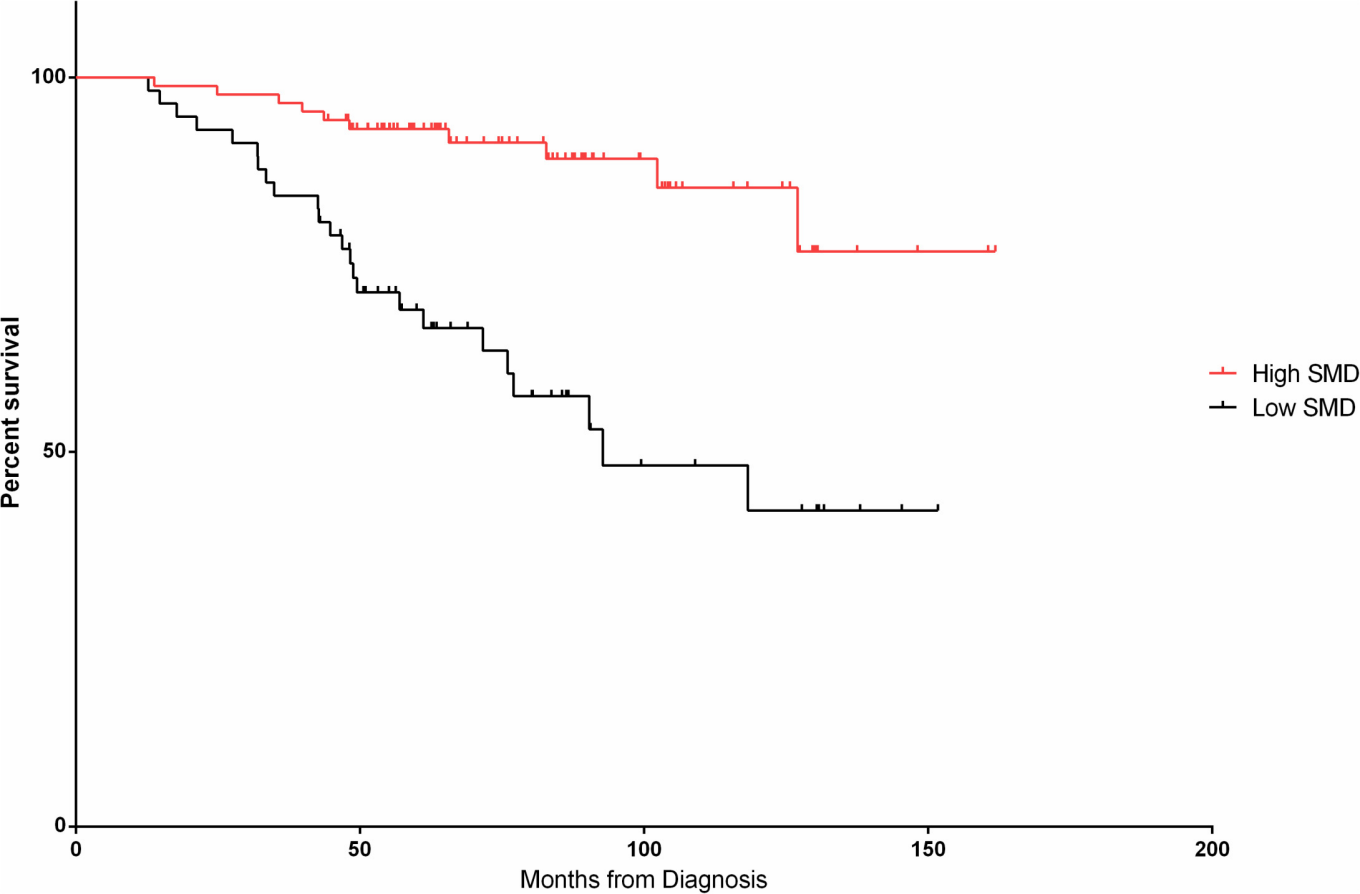
Overall survival (OS) along SMD cut-point.

After adjusting for FLIPI-1 scores and gender in multivariate Cox proportional hazards modeling (Table 2), low SMD showed a strong trend towards poorer PFS (HR 1.65, 95% CI 0.98–2.79, *p* = 0.06, see Fig 1). Low SMD was still associated with poorer OS independent of FLIPI-1 and gender (HR 3.40, 95% CI 1.58–7.32, *p* = 0.002, see Fig 2). Despite a significant discrepancy in age between high and low SMD groups, FLIPI-1 already accounts for age in its scoring system [24]. Therefore, age was not included in multivariate analyses to avoid using the variable twice.

**Table 2:** Univariate and multivariate analyses of variables assessing for impact on progression free survival (PFS) and overall survival (OS); 95% CI = 95% confidence interval, FLIPI-1 = Follicular Lymphoma International Prognostic Index 1, SMD = Skeletal Muscle Density.

	Variable	Univariate Hazard Ratio	95% CI	*p*-value	Multivariate Hazard ratio	95% CI	*p*-value
PFS	FLIPI-1 score 2	1.24	0.62–2.46	0.54	1.59	0.67–2.61	0.44
	FLIPI-1 score 3	2.31	1.17–4.55	0.01	1.74	0.79–3.81	0.17
	FLIPI-1 score 4–5	2.01	0.94–4.30	0.07	2.09	1.05–4.16	0.03
	Gender, male	1.41	1.05–1.89	0.02	1.21	0.73–2.01	0.46
	Low SMD	1.85	1.13–3.03	0.01	1.65	0.98–2.79	0.06
OS	FLIPI-1 score 2	1.28	0.44–3.68	0.65	1.46	0.50–4.22	0.49
	FLIPI-1 score 3	2.55	0.94–6.89	0.06	2.21	0.81–6.00	0.12
	FLIPI-1 score 4–5	3.83	1.39–10.56	0.01	2.76	0.97–7.82	0.06
	Gender, male	1.21	0.62–2.38	0.57	1.23	0.62–2.44	0.55
	Low SMD	4.02	1.93–8.37	0.0002	3.40	1.58–7.32	0.002

### Skeletal Muscle Index

As a cohort, median SMI was 55.8 cm^2^/m^2^ in males and 43.2 cm^2^/m^2^ in females. Reduced muscle surface area did not impact OS in spite of using sarcopenia definitions as outlined in solid organ malignancies by Prado *et al*. [2] (HR 1.26, 95% CI 0.75–2.12, *p* = 0.37); as outlined by Lanic *et al*. for DLBCL [18] (HR 1.41, 95% CI 0.86–2.32, *p* = 0.17); or as set out by Martin *et al*. [4] (HR 1.21, 95% CI 0.53–2.79, *p* = 0.65). Cut-point analysis did not yield a discernible muscle index definition related to OS outcomes (HR 1.06, 95% CI 0.60–1.87, *p* = 0.84). Effects on PFS using all four definitions of sarcopenia yielded similar findings.

### Response to chemoimmunotherapy

As a secondary endpoint, both SMI and SMD were examined for affects on radiographic response to chemoimmunotherapy by Cheson criteria [23]. Higher ORR was more associated with higher SMD. 96% of patients with a high SMD had at least a partial response (PR) as compared to 83% in the lower SMD group (*p* = 0.01). SMI was not associated with response.

## Discussion

FLIPI-1 score and Ann Arbor staging systems recognized the importance of age and weight loss, respectively [9, 24]. It was not until emergence of CD20-directed therapy that gender became recognized as a prognostic factor [16]. Lanic *et al*. [18] sought to identify underlying mechanisms of age-dependent outcomes and noted that sarcopenia in older DLBCL patients may be the cause of poorer outcomes.

However, our findings in an unselected FL population find higher SMD measured by CT is associated with improved PFS and OS. This PFS improvement is in spite of the lower SMD group having a higher use of maintenance rituximab (Table 1) [25]. The substantial OS difference is interesting considering that this study did not track second or third line therapy including some patients who may have continued onto high-dose therapy and autologous stem cell transplantation. In this way, our findings suggest that pre-treatment SMD measurements may be an independent prognostic marker of FL patient outcomes. However, higher pre-treatment SMD was associated with improved ORR and PFS—raising the question as to whether SMD is in fact a prognostic indicator or a predictor for response to treatment. Though looking at treatment completion CT images to examine for improvement in SMD following chemoimmunotherapy was of interest, the heterogeneity of CT scan timing at treatment end led to its exclusion from this retrospective review.

Further, SMD carried more prognostic weight than individual FLIPI-1 (including advanced age) points despite factoring in FLIPI-1 scores and gender in multivariate analysis between high and low SMD groups [24]. Though FLIPI-2 was validated in the rituximab era, FLIPI-1 remains a widely used measure of prognosis in FL patients due to its ease of use [26]. Though additional research software was used to extract the data considered here, CT scans used to measure SMD were CT scans done on standard clinical practice CT scanners as part of routine care. Standard CT imaging can measure radiation attenuation (though not average radiation attenuation as measured here), and does not require additional equipment or software to obtain this value. If SMD were to be validated as a prognostic marker, incorporation of a function to measure average radiation attenuation into CT imaging software could be easily done. For that reason, SMD presents a potentially easy and inexpensive method of better stratifying patients to complement FLIPI-1 in the rituximab era.

Despite using skeletal muscle index parameters defined in multiple tumor subtypes, sarcopenia does not appear to impact FL patient outcomes as strongly as in DLBCL. Perhaps due to FL’s indolent nature, skeletal muscle mass is not as sensitive in prognosticating survival and does not explain differences seen across age and gender subsets. However, sarcopenia’s lack of impact on survival outcomes may lend further support to the theory that differing outcomes in these subsets is more related to rituximab dose [16].

It should be noted that SMD cut-off parameters between non-overweight and overweight patients is quite small at 36.6 vs. 33.1 HU as compared to those identified by Martin *et al*. [4]. These cut-off values are not that dissimilar (and the lower value in overweight patients essentially equal to the threshold identified by Martin *et al*.) from those identified in solid tumors and the slight discrepancy likely reflects our smaller patient cohort. Specifically, only forty patients were identified as non-overweight by BMI that makes validating our SMD cut-off in that specific population somewhat difficult. With more patient numbers, it may have been possible to validate Martin *et al*.’s definitions in follicular lymphoma given the trend towards significance in PFS (*p* = 0.18). Naturally, it would be helpful to validate our findings in a prospective fashion, or in patients treated with most centers’ new standard of care, bendamustine and rituximab. However, because bendamustine and rituximab was only widely adopted in Canada after its national approval in 2012, it will be difficult to properly correlate PFS and OS due to the lack of follow-up time. In the original phase III trial, the median PFS for patients on this combination was nearly 70 months [13].

Why SMD has such a strong link to FL response to chemoimmunotherapy and outcomes is a matter for speculation. Higher SMD is associated with improved survival in melanoma, renal cell cancer, and a number of solid tumors [4–6]. Perhaps SMD is a more accurate measure of muscle function and therefore precedes sarcopenia development. It could very well be that reduced muscle density is a further manifestation of muscle wasting, in the sense that the lower the mean density, the lower the amount of muscle within the accepted normal range (+30 to 150 HU). In this way, low SMD may be a more sensitive method of detecting patients who are more ill than sarcopenia—objectifying a process that clinicians try to determine with performance status assessments.

Inflammation is a potential mechanism for both fatty infiltration of muscle and type 2 diabetes [7]. Higher intramuscular adipose tissuewas strongly associated with insulin resistance and higher serum inflammatory markers including C-reactive protein, interleukin-6, and tumor necrosis factor-alpha. Intramuscular adipose tissue is an absolute measure of adipose tissue in muscle and, as such, is related to SMD. However, SMD is more strongly correlated with overall muscle quality [21]. Therefore, in malignancies, SMD may be a reflection of increased production of multiple pro-inflammatory cytokines. Data on such cytokines was not available in this retrospective review, but is a matter of interest for future study.

Other limitations of this study include the inherent bias introduced by a retrospective analysis. Consequently, though SMD was more predictive of outcomes than FLIPI-1, it may be helpful to validate these findings in a prospective study. Further as a retrospective study, physical functional assessments were not collected, but neither are they typical in medical oncology. FLIPI-2 was not used as a result of the lack of β2-microglobulin testing. R-CVP was induction treatment of choice for this cohort. Within the defined study dates, bendamustine-rituximab was newly presented and not yet approved in Canada [13]. R-CVP was also the institutional standard given that it had a favorable toxicity profile and performed no differently than other investigated combinations [11, 27].

Future directions incorporate validating SMD prospectively as a prognostic or predictive tool in FL patients alongside determining whether SMD changes with chemoimmunotherapy use. Given a possible link between SMD and inflammation, another possibility includes monitoring serial serum inflammatory markers to determine a link between specific cytokines in patients with low SMD. Further investigation is underway as to whether SMD changes following completion of chemoimmunotherapy and its applicability in other lymphoma subtypes such as diffuse large B-cell and mantle cell lymphoma.
